# High adiponectin levels in lean Arab women compared to Asian women

**DOI:** 10.1186/s40364-015-0032-5

**Published:** 2015-04-11

**Authors:** Mohamed Abu-Farha, Kazem Behbehani, Naser Elkum

**Affiliations:** Biochemistry and Molecular Biology Unit, Dasman Diabetes Institute, Kuwait City, Kuwait; Dasman Diabetes Institute, Kuwait City, Kuwait; Clinical Epidemiology, Sidra Medical and Research Center, 26999, Doha, Qatar

**Keywords:** Adiponectin, Ethnicity, Obesity, BMI, Arab, Asian

## Abstract

Adiponectin has been recognized as a potent regulator of metabolism possessing anti-inflammatory and anti-atherogenic functions and inversely associated with increasing incidents of type 2 diabetes and obesity. In this study, we investigated the changes in adiponectin level of 193 Arab and 132 Asian women were compared. Overall, Arab women had statistically significant higher levels of adiponectin 17.84 (1.047) μg/mL than Asians 12.87 (1.049) μg/mL. In conclusion, our data demonstrates that Arab women poses high adiponectin level compared to Asians and the protective role of adiponectin in Arab women against metabolic disorders requires further attention.

## To the Editor

Adiponectin has been recognized as a potent regulator of metabolism possessing anti-inflammatory and anti-atherogenic functions. Its level in human plasma is inversely associated with increasing incidents of type 2 diabetes, cardiovascular disease risk and obesity [1]. Significantly different levels of adiponectin have been reported in different ethnicities. For example, Asians have a lower adiponectin plasma level than Caucasians, which is consistent with the increased risk of metabolic disorders among Asians [1].

In this study, plasma level of adiponectin of 193 Arab and 132 Asian women living in Kuwait was assessed using the multiplexing immunobead array platform (Luminex, Austin, TX) using a 2-Plex kit (BioRad, Hercules, CA). All subjects gave their written consent to participate in the study. Full description of the study design was published by Elkum et al. [2]. Briefly, our samples were collected from the six governorates of the state of Kuwait, where random samples were collected from each stratum with proportional allocations. Adiponectin level are reported as geometric means and their standard errors according to BMI. BMI between 18.5 and 24.9 was considered lean, 25 to 29.9, overweight, ≥30 was considered obese). Clinical characteristics of the population are shown in Table 1.

**Table 1:** **Clinical and biochemical profile for Arabs and Asians**

**Variables**	**Lean N = 18**	**Overweight N = 49**	**Obese N = 126**
**Arabs**			
FBG (mmol/l)	4.87 ± 0.54	5.26 ± 1.77	6.35 ± 2.76
Total cholesterol (mmol/l)	5.00 ± 0.99	5.20 ± 1.28	5.40 ± 0.99
Triglyceride (mmol/l)	1.02 ± 0.47	1.43 ± .68	1.66 ± 0.89
LDL cholesterol (mmol/l)	3.05 ± 0.99	3.35 ± 1.22	3.49 ± 0.89
HDL cholesterol (mmol/l)	1.54 ± 0.59	1.26 ± 0.30	1.21 ± 0.38
Systolic (mmHg)	109.06 ± 10.23	117.94 ± 15.15	129.72 ± 20.25
Diastolic (mmHg)	66.33 ± 10.15	73.96 ± 8.98	79.53 ± 13.07
**Asians**			
**Variables**	**Lean N = 51**	**Overweight N = 48**	**Obese N = 33**
FBG (mmol/l)	4.99 ± 1.42	5.05 ± 2.62	5.26 ± 1.34
Total cholesterol (mmol/l)	4.99 ± 1.15	5.09 ± 0.95	5.05 ± 0.95
Triglyceride (mmol/l)	1.02 ± 0.52	1.40 ± 0.88	1.45 ± 0.71
LDL cholesterol (mmol/l)	2.99 ± 0.99	3.07 ± 0.82	3.13 ± 0.83
HDL cholesterol (mmol/l)	1.56 ± 0.33	1.26 ± 0.26	1.3 ± 0.30
Systolic (mmHg)	122.82 ± 18.44	122.00 ± 19.15	136.00 ± 21.30
Diastolic (mmHg)	74.31 ± 9.21	79.00 ± 11.34	80.00 ± 13.77

**Figure 1:**
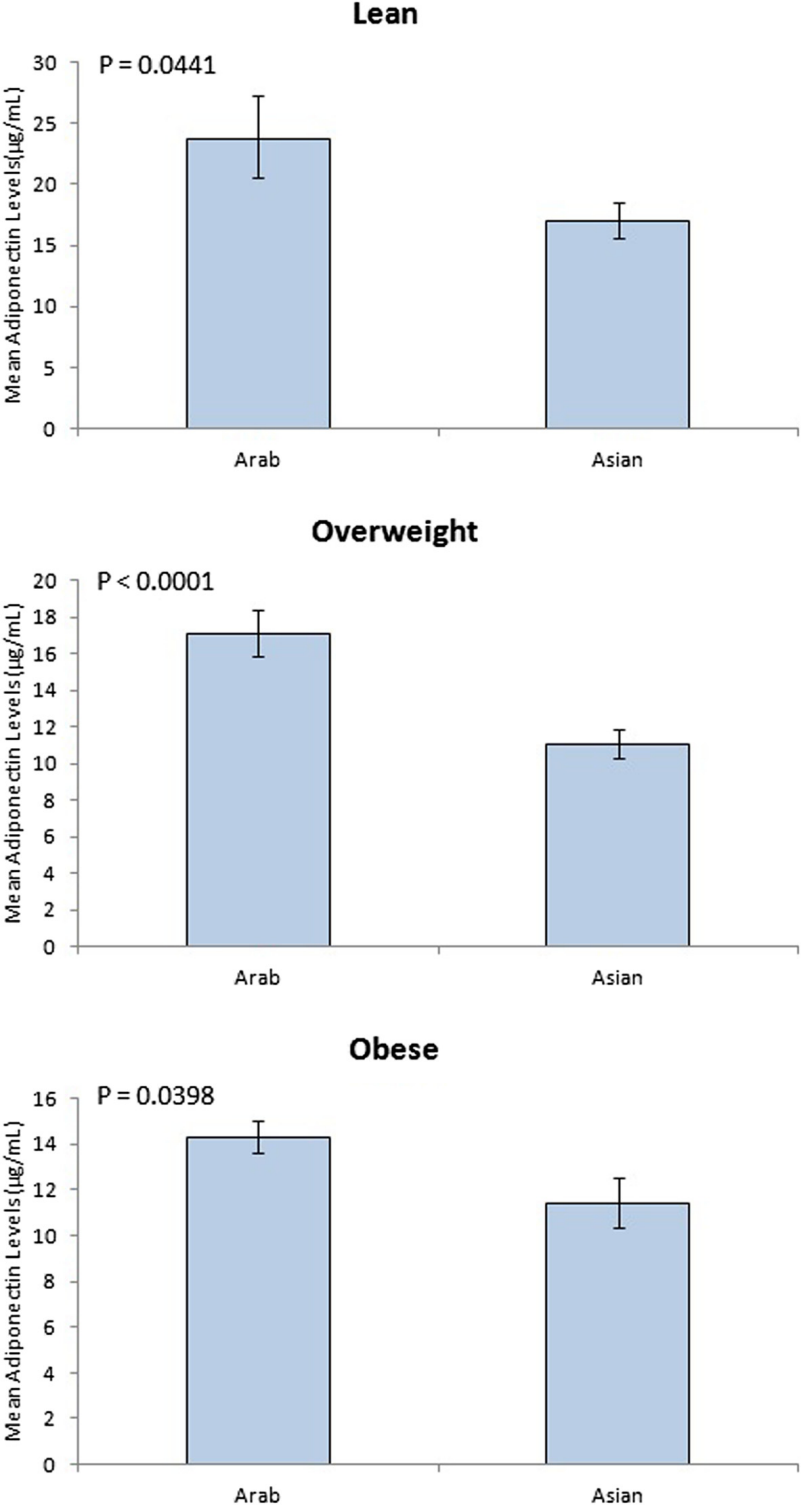
**Adiponectin level in Arab and Asian women.** Ethnic difference in adiponectin level between Arab and Asian women at different BMIs. BMI between 18.5 and 24.9 was considered lean, 25 to 29.9, overweight, ≥30 was considered obese.

Overall, Arab women had statistically significant higher levels of adiponectin 17.84 (1.047) μg/mL than Asians 12.87 (1.049) μg/mL. Comparing across different levels of BMI, Arab lean women had the highest adiponectin level at 23.73 (1.15) μg/mL; overweight women, 17.10 (1.08) μg/mL; and obese, 14.30 (1.06) μg/mL. On the other hand, adiponectin level in lean Asians was 17.00 (1.09) μg/mL, overweight 11.07 (1.07) μg/mL, while obese women had 11.42 (1.10) μg/mL Figure 1.

The significance of this study is that it compared cohorts of Arab and Asian women residing in Kuwait. The adiponectin levels for Asians reported in this study match with those reported in other studies using Asian samples such as Mente et al. (Adiponectin 11.02 ± 0.76 μg/mL, BMI 26.1 ± 0.3 Kg/m^2^) [1] and Smith et al. (Adiponectin 10.73 ± 1.35 μg/mL, BMI 28.7 ± 0.86 Kg/m^2^) [3]. Our data shows that Arab women have a significantly higher adiponectin level than Asians. Arab women might also have higher levels of adiponectin compared to women from other ethnicities such as Hispanic (11.7 μg/mL), Caucasians (15.6 μg/mL), far-East Filipinos (8.9 μg/mL) and African Americans (9.67 μg/mL) [4].

Caucasians have the highest reported adiponectin levels out off all ethnicities. Using an Asian population as a bench mark, adiponectin levels in lean Arab women might be higher than those in Caucasians. Araneta et al. reported that lean Caucasian women had a mean adiponectin level of 15.66 μg/mL, which is significantly lower than the 23.73 μg/mL concentrations reported in the current study [4].

In conclusion, our data shows that lean Arab women have a high adiponectin levels compared to Asians and potentially other ethnicities especially Caucasians. In the future, large cohort studies involving other ethnicities, mainly Caucasians, alongside Arabs will be beneficial to further demonstrate the role of adiponectin in this population.
